# Prevalence of Some Genetic Risk Factors for Nicotine Dependence in Ukraine

**DOI:** 10.1155/2019/2483270

**Published:** 2019-10-20

**Authors:** Vitalina Bashynska, Alexander Koliada, Kateryna Murlanova, Oksana Zahorodnia, Yuliia Borysovych, Vladyslav Moseiko, Oleh Lushchak, Alexander Vaiserman

**Affiliations:** ^1^Diagen Genetic Laboratory Limited, Kyiv, Ukraine; ^2^Chebotarev State Institute of Gerontology, Kyiv, Ukraine; ^3^Vasyl Stefanyk Precarpathian National University, Ivano-Frankivsk, Ukraine

## Abstract

Tobacco smoking is known to be a strong risk factor for developing many diseases. The development and severity of smoking dependence results from interaction of environmental and lifestyle factors, psycho-emotional predispositions, and also from genetic susceptibility. In present study, we investigated polymorphic variants in genes contributed to nicotine dependence, as well as to increased impulsivity, known to be an important risk factor for substance use disorders, in Ukraine population. The genotype frequencies at *CYP2A6*, *DNMT3B*, *DRD2*, *HTR2A*, *COMT*, *BDNF*, *GABRA2*, *CHRNA5*, and *DAT1* polymorphisms were determined in 171 Ukraine residents, and these data were compared with data for several other European populations and main ethnic groups. It has been found that genotype frequencies for all studied loci are in Hardy-Weinberg equilibrium in the Ukrainian population and correspond to the respective frequencies in European populations. These findings suggest a similar impact of these loci on nicotine dependence in Ukraine. Further studies with larger sample sizes are, however, needed to draw firm conclusions about the effect size of these polymorphisms.

## 1. Introduction

Tobacco smoking is an important risk factor for developing many diseases, including cardiovascular, broncho-pulmonary, oncological, and psychiatric disorders [1]. Nicotine dependence, like other substance use disorders (SUDs), is a highly complex phenotype. The development and severity of smoking dependence results from interaction of various environmental and lifestyle factors, psycho-emotional predispositions, and also from polygenic susceptibility [2]. Data from twin studies worldwide have demonstrated that 33–71% of the variation in liability to nicotine dependence may be attributed to heritable influences [3]. The differences in heritability estimates obtained in these studies may be likely attributed to differences in diagnostic criteria used, in particular, in measures of quantity/frequency (cigarettes smoked per day, etc.,) [3]. According to findings from these studies, genetic and environmental factors play significant and approximately equal roles in determining both smoking initiation and persistence. For example, a meta-analysis of 16 studies across different countries have indicated that both these phenotypes are substantially heritable [4]. Across these studies, the heritability of smoking initiation was shown to be 37% for men and 55% for women, while the heritability of smoking persistence was 59% and 46% for men and women, respectively. These data suggest that genetic and environmental factors can contribute differently to the determination of smoking initiation and persistence in male and female smokers. In particular, genetic factors may play a more significant role for smoking initiation but a less significant role for smoking persistence in female compared to male adults. In addition, genetic susceptibility may contribute differently to SUDs development in different age periods. In particular, while shared environmental factors contribute maximally to familial resemblance during adolescence, hereditary factors account for up to 75% of individual differences in adulthood [5]. Genes responsible for susceptibility to smoking dependence may act through changes in drug metabolism caused by particular nicotine metabolic gene variants and via modified functioning of the nicotinic receptor, which can change both affinity for nicotine and circuitry of reward. Furthermore, they may influence general mechanisms of addiction. In particular, genes such as monoamine oxidase A and the serotonin transporter may modulate stress response, and also emotion and behavioral control [2]. The candidate gene variants include cholinergic nicotine receptor genes such as *CHRNB3* and *CHRNA5*, dopamine pathway genes (*DRD2* and *DRD4*), dopamine transport (*DAT1*) genes, serotonin pathway genes such as tryptophan hydroxylase, *TPH* (associated with serotonin biosynthesis) and serotonin transporter, *5HTTLPR* (associated with serotonin reuptake) genes, monoamine oxidase (*MAO-A*) and dopamine beta hydroxylase genes affecting norepinephrine pathways, and also genes involved in the metabolism of nicotine such as *P540 CYP2A6* [2]. Findings from different populations are, however, contradictory: the loci, associated with smoking, as well as size and direction of the allelic effects vary in different studies, suggesting the need for replication research in different regional, ethnic and sociocultural settings.

In Ukraine, tobacco smoking is an important public health issue today. According to the age-standardized prevalence estimates for daily tobacco smoking among persons aged 15 years and above in Ukraine in 2015, daily tobacco smoking prevalence was 43.0% in males and 10.2% in females [6]. The study of genetic variations underlying tobacco addiction has, however, never been done before in Ukraine population. In present research, we estimated allelic and genotypic frequencies at polymorphic loci associated with nicotine dependence and/or with the risk factor for an addictive behavior such as impulsivity in Ukraine, and these data were compared with data for several other countries and ethnic groups in Europe.

## 2. Materials and Methods

The study included 171 volunteers (141 women, 30 men) living in Ukraine, with no psychiatric diagnoses, mean age 32.6 ± 9.6 years. All participants gave informed consent and donated buccal epithelium samples for DNA extraction. The single nucleotide polymorphisms (SNPs) in the genes *DNMT3B *rs910083*, DRD2 *rs1800497* (TaqI), HTR2A *rs6313*, CYP2A6 *rs4105144*, COMT *rs4680 (Val158Met),* BDNF *rs6265*, GABRA2 *rs279858*, CHRNA5 *rs16969968и* DAT1 rs28363170 *(VNTR в 3`UTR) were determined. The selected loci were genotyped using allele-specific PCR or PCR with analysis of restriction or amplification fragment length polymorphism (RFLP/AFLP). The genotyping methods used in the study are presented in Table 1.

The genotype frequencies at the studied loci in Ukraine population were compared with other European populations as well as with non-European populations, using data from 1000 Genomes [8] and HapMap [9] projects. Deviations in genotype frequencies from Hardy–Weinberg equilibrium as well as significance of differences in genotypes distribution between populations were assessed using the *χ^2^* test. When any of the genotype frequencies were zero, then the comparison was performed using Fisher's two-sided test. Differences were considered significant when the* χ^2^* criterion exceeded the critical value for (*n* − 1) degrees of freedom, where *n* was the number of genotypes taken into account, at significance level 0.05. For the loci with zero genotype frequencies, differences with *p* < 0.05 were considered significant.

## 3. Results and Discussion

**Figure 1:**
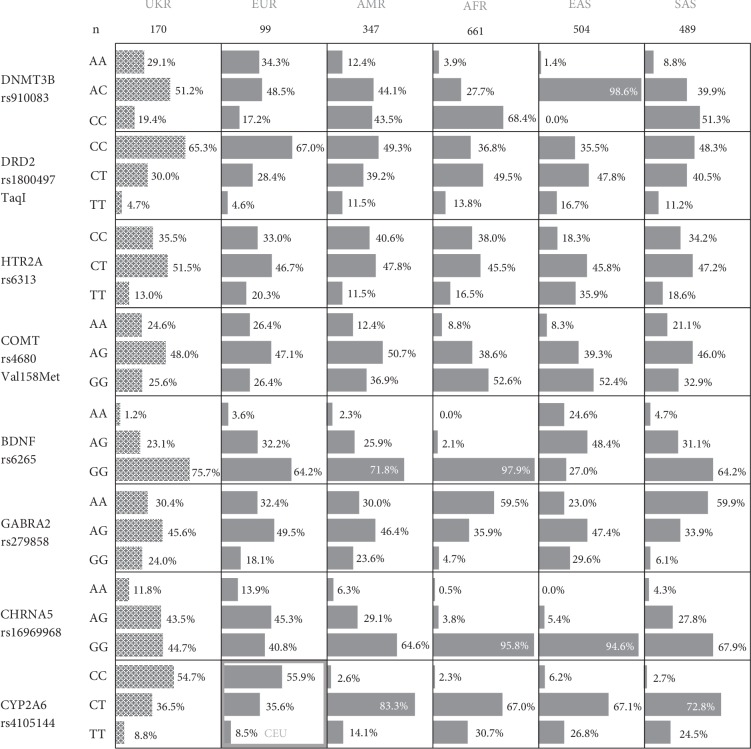
Genotype frequencies for *DNMT3B* rs910083, *DRD2* rs1800497, *HTR2A* rs6313, *CYP2A6* rs4105144, *COMT* rs4680, *BDNF* rs6265, *GABRA2* rs279858, and *CHRNA5* rs16969968 loci. UKR—Ukrainian population. EUR—Europeans, CEU—Central European population (Utah, USA), AMR—Ad Mixed Americans (South and Latin America), AFR—Africans and African Americans, EAS—East Asians, SAS—South Asians (according to the data from the 1000 Genomes project).

Genotype frequencies for all studied loci were in Hardy-Weinberg equilibrium (data not shown).  Figure 1 represents genotype frequencies at the biallelic loci in Ukrainian population and superpopulations analyzed in 1000 Genomes project (European, Ad Mixed American, African, East and South Asian).

As it is evident from the Figure 1, genotypes at the studied loci are distributed in Ukraine similarly to European populations. The only significant *χ^2^* value has been shown for *BDNF* rs6265, which has rare minor homozygous genotype in all ethnic groups. We suppose that for more reliable comparisons of genotypic frequencies, further recruiting of sample is required: in case of rare alleles/genotypes, a minor skew in frequencies in small groups can lead to false positive conclusions on differences in genotypes distributions. For the *HTR2A*, *BDNF*, and *GABRA2* genes, the genotypes are distributed in Ukraine similarly to admixed American populations as well.

In the HapMap and 1000 Genomes projects, controversial results were obtained for allelic frequencies of the CYP2A6 rs4105144 within the Central European population. This SNP lies in the region of high homology with nearby genes, and a number of minor alleles in these regions can be misinterpreted as a minor T allele at rs4105144 [10]. Based on this, we compared our data with those for the Central European population in the HapMap project, where lower frequencies of T allele and CT and TT genotypes were obtained. Genotype frequencies at rs4105144 in Ukraine are in accordance with these data, but the genotyping issues require validation of these results by other methods.

Keeping in mind the notable heterogeneity of the superpopulations analyzed in the 1000 Genomes Project, we conducted a more detailed comparison of our results for the biallelic loci with those for the European populations that constitute the European supergroup.  Table 2 shows genotype frequencies for Ukrainian and 1000 Genomes European populations along with *χ*^2^ and p-values for their difference with our data. As can be seen from Table 2, each of the SNPs, including *BDNF* rs6265, has genotype frequencies in Ukraine comparable to at least one of the European populations. For *CYP2A6*, such a comparison was not performed due to the abovementioned clearly overestimated T allele frequencies in populations in the 1000 Genomes project and the lack of such data for other European populations, except for the Central European one, in the HapMap project.

All the genes analyzed in our study are involved in the molecular mechanisms of SUDs pathogenesis. In particular, CYP2A6 is the main enzyme metabolizing nicotine. Many studies have shown the association of the activity of this enzyme with smoking status and intensity [11–14]. We selected the rs4105144 SNP in our study instead of the polymorphisms tagging the low-activity enzyme variants. The rs4105144 is associated with CYP2A6 activity, though with less effect size, and its minor allele is significantly more frequent than the main poor-metabolizing alleles of CYP2A6 [11], that is advantageous for genetic association studies in case of inadequate power. This polymorphism is associated with smoking according to the data of genome-wide association and candidate gene studies, however, due to the already mentioned difficulties of genotyping, the results require validation by independent methods. However, the frequencies of the TT smoking risk genotype (8.8%) and of the C protective allele (72.9%) in our study are in accordance to the frequencies obtained in samples of Europeans in which the association was shown [13, 14].

DNMT3B is a DNA methyltransferase 3 beta that is responsible for *de novo* methylation during embryogenesis and maintaining methylation in adulthood [15]. The carriage of the risk allele for nicotine dependence, rs910083∗C, is associated with increase in DNMT3B mRNA expression in the cerebellar cortex [16]. Experimental data indicate an increase in the level of DNMT3B expression and DNA hypermethylation during smoking [17], as well as the association of rs910083∗C with squamous cell lung carcinoma, which makes DNMT3B one of the possible targets for nicotine dependence therapy [16]. The C allele and CC genotype carriage frequencies in Ukrainians are 70.6% and 19.4%, correspondingly, in line with data for the Central European and British populations, as well as to the European group in a whole.

*DRD2* gene encodes the D2-receptor for dopamine. This receptor is expressed in large quantities in the main areas of dopamine projection (including the nucleus caudate, putamen, nucleus accumbens and the olfactory tubercle), in the body cells of dopaminergic neurons in the substantia nigra and in the ventral tegmental area [18]. In carriers of the risk allele for substance dependence rs1800497∗T, there is a decrease in the density of D2 receptors by 40% compared to homozygotes for allele C [19], this can lead to an “understimulated” state that can be alleviated by smoking and/or using other psychoactive substances [20]. The carriage frequency of the T allele in our sample is 34.7%; the distribution of allele and genotype frequencies for this locus corresponds to all European populations except for Spanish.

*HTR2A* encodes the serotonin receptor type 2A (5-HT2A). It also functions as a receptor for various drugs and psychoactive substances, including psilocybin and LSD [21]. Activation of this receptor affects perception, cognitive abilities and mood, plays a role in the regulation of behavior, including reactions to anxiogenic situations [22]. The rs6313∗C allele carriage is associated with decreased expression of 5-HT2A receptors. In different studies, either C or T alleles were associated with smoking status and intensity [23, 24]. The frequency of rs6313∗TT genotype in Ukraine is 11.3%, the data correspond to the Finnish population, as well as to mixed groups of European, South American, African, and South Asian origins.

Catechol-O-methyltransferase (COMT) is an enzyme that plays an important role in the breakdown of catecholamines such as dopamine, adrenaline, and norepinephrine. The “Worrier or Warrior” rs4680 (Val158Met) polymorphism selected in this study correlates with enzyme activity and, accordingly, with dopamine levels. This polymorphism is associated with impulsivity level, stress resilience, and addiction to psychoactive substances [25, 26, 27]. The GG genotype, associated with lower smoking intensity and more effective smoking cessation [26, 28], was revealed in Ukraine population at frequency 25.6%; the genotypes at this locus are distributed in Ukrainians similarly to all European populations except the Finnish.

The brain-derived neurotrophic factor, BDNF, is necessary for growth, differentiation, and maintenance of dopaminergic and other neurons. The rs6265∗A allele carriage correlates with reduced expression of BDNF in response to stimulation by psychoactive substances in several brain areas, including hippocampus. This can have a negative effect on short-term memory [29, 30, 31] and is negatively associated with the initiation of smoking [32, 33]. In addition, this allele is associated with increased stress reactivity and anxiety [34], and the GG genotype is associated with depression [35]. Allele A carriage frequency was 24.3% in Ukraine; in general, alleles and genotypes at this locus were found to be distributed similarly to those in the British, Finnish, and Ad Mixed American populations.

*GABRA2 *gene encodes a gamma-aminobutyric acid receptor, an inhibitory neurotransmitter that regulates serotoninergic signal transduction in the central nervous system. Carriage of G allele at rs279858 investigated in our study is associated with increased risk of nicotine, alcohol, and heroin addictions [36–38]. A possible neurobiological mechanism of this genetic association is lower neural connections' (connectome) efficiency in the reward system in GG carriers compared to carriers of the protective A allele [38], which is, in turn, due to reduced GABRA2 mRNA expression [39]. The frequency of the rs279858∗GG genotype in Ukraine (24.0%) is in line with data for ad-mixed European and American groups, as well as for the Central European and British populations.

*CHRNA5* gene encodes the neuronal acetylcholine receptor subunit alpha-5 (nAChR*α*5), which constitutes nicotinic cholinergic receptors both in the CNS and in the peripheral ganglia. Nicotine can act as one of the agonists of nAChR receptors. The smoking risk allele, rs16969968∗A [40] results in nAChR*α*5 variant with reduced permeability to Ca2+ and greater desensitization (i.e. reduced functionality) compared to the wild-type receptor isoform (G allele carriers) in presynaptic nAChRs in the CNS [41]. The AA genotype at this locus confers increased risk for lung cancer [42]. This genotype frequency in Ukraine is 11.8%, this corresponds to the admixed European and Central European populations.

**Figure 2:**
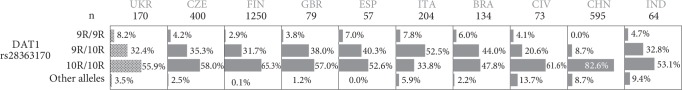
Frequency distribution of genotypes at *DAT1* rs28363170 (%) in Ukrainian and other populations according to literature data [43, 47–53]. UKR–Ukrainians, CZE – Czechs, FIN—Finns (male only), GBR—UK residents of European descent, ESP—Spaniards, ITA—Italians, BRA—Brazilians of European descent, CIV—Ivory Coast residents, CHN—Han Chinese population, IND—Indians (Badaga population).

DAT1 is a dopamine transporter gene involved in dopamine reuptake from the synaptic cleft into presynaptic neuron. Unlike other polymorphisms selected in this study, *DAT1* rs28363170 is a variable number of tandem repeats polymorphism (VNTR) in the 3′-UTR of the gene and is characterized by repetition alleles with 3–16 repeats of the 40-bp segment [43]. 9 and 10 repeats are the most common for this VNTR. Various number of repeats in the 3′UTR region of this gene may affect its expression level; however, various *in vitro* studies gave inconsistent data; however, 10R was shown to act as a transcriptional enhancer [43, 53]. At the same time, individuals with 10R/10R genotype were shown to have decreased efficiency of binding to the dopamine transporter in striatum when analyzed by single positron emission computed tomography [53, 55]. Carriage of 9 repeats is associated with alcohol dependence [53] and with increased impulsivity and emotionality [44], the traits that are risk factors for addictions to nicotine and other psychoactive substances [45, 46]. Due to genotyping issues, there are no data on minor alleles and genotypes frequencies in large-scale projects aimed at genotyping of thousands of people mainly on single-nucleotide polymorphisms. Therefore, we compared distributions of genotypes in Ukraine and in other countries using literature data [43, 47–53]. The results are presented in Figure 2. As in the vast majority of populations, the most frequent genotypes in Ukrainians are 10/10 (55.9%) and 9/10 (32.4%); on the whole, the distribution of genotypes in our sample does not differ significantly from the data for the Czech Republic [47] and Spain [50].

## 4. Conclusion

Identification of genetic factors predisposing to nicotine addiction or to psychotypes leading to the use of psychoactive substances contributes to a better understanding of the molecular mechanisms of addiction and to the emergence of personalized therapeutic approaches. In addition, one of the modern concepts of working with addictions is the idea of reducing the harm from psychoactive substances. It becomes clear that approaches based on harm reduction concepts are particularly relevant for people with a strong genetic risk factor burden that needs to be studied in various ethnic groups.

Comparison of genotype frequencies at the selected polymorphic loci in Ukraine with the data for other populations showed that SUD risk alleles and genotypes, including smoking dependent risk variants, are presented in the Ukrainian population with frequencies similar to those in other European populations. The percentage of adult people who constantly or rarely used tobacco, ranged from 14.8% to 46% in 2016 with average of 28.7% for European countries and with worldwide average of 21.9%, according to the WHO data [54]. In general, data on the prevalence of smoking in Ukraine and in the majority of European countries with similar frequencies of the abovementioned smoking risk alleles do not differ significantly and lie within the range of 20–30%. Based on this, we can assume that the studied genetic polymorphisms may have an impact on the smoking risk and prevalence in Ukrainian population, similar to that in other European ethnic groups. However, to estimate the effect of these loci, a significant increase in the sample size and association analysis is required.

## Figures and Tables

**Table 1:** Primer sequences used for genotyping the selected loci.

Localization	Gene	SNP, allele1/allele2	Genotyping method
4p12	*GABRA2*	rs279858, A/G	Allele-specific PCR. Primers: 5′-TGGAATTTTACCGTGTCTTGTGTC-3′, 5′-ACAGCTAGATTGGCTGGTTGTT-3′, 5′-ATATTATGAGCTACTGATTTT-3′, 5′-ATATTATGAGCTACTGATTTC-3′

5p15.33	*DAT1*	rs28363170 (VNTR), 3-16 repeats	AFLP-PCR. Primers: 5′- TGCGGTGTAGGGAACGGCCTGAG-3′, 5′-CTTCCTGGAGGTCACGGCTCAAGG-3′

11p14.1	*BDNF*	rs6265, A/G	Allele-specific PCR, according to [7]

11q23.2	*DRD2*	rs1800497, C/T	PCR-RFLP. Primers: 5′-GGCTTAGAACCACCCAGAGT-3′, 5′-GAGCACCTTCCTGAGTGTCA-3′ Restriction endonuclease *Taq I*

13q14.2	*HTR2A*	rs6313, C/T	Allele-specific PCR. Primers: 5′-TACAGTAATGACTTTAACTCC-3′, 5′-TACAGTAATGACTTTAACTCT-3′, 5′-AAGGAGAGACACGACGGTGA-3′

15q25.1	*CHRNA5*	rs16969968, A/G	Allele-specific PCR. Primers: 5′-TGGGGGAAGTGGAGAAGTGA-3′, 5′-ACATTGGAAGCTGCGCTCA-3′, 5′-ACATTGGAAGCTGCGCTCG-3′

19q13.2	*CYP2A6*	rs4105144, C/T	Allele-specific PCR. Primers: 5′-CAGATATCAATCGCTCTAATCCT-3′, 5′-CAGATATCAATCGCTCTAATCCC-3′, 5′-TTCCTAGTGCCAACCCCAGT-3′

20q11.21	*DNMT3B*	rs910083, A/C	PCR-RFLP. Primers: 5′-GGAGACTAGAACATTCCCTGTGT-3′, 5′-GGCCATTCAGGGATGCTAATC-3′ Restriction endonuclease *Sal I*

22q11.21	*COMT*	rs4680, A/G	Real-time allele-specific PCR with the use of genotyping kit produced by LLC scientific and production company “LYTECH”

**Table 2:** Genotype frequencies at the biallelic polymorphic loci in Ukraine and the European populations analyzed in 1000 Genomes project.

Gene, SNP	Genotype	Ukraine, *N* (%)	Central Europe, *N* (%)	Finland, *N* (%)	Great Britain, *N* (%)	Spain, *N* (%)	Italy, *N* (%)
*DNMT3B* rs910083	AA	50 (29.4%)	34 (34.3%)	30 (30.3%)	28 (30.8%)	45 (42.1%)	37 (34.6%)
	AC	87 (51.2%)	48 (48.5%)	42 (42.4%)	41 (45.1%)	48 (44.9%)	57 (53.3%)
	CC	33 (19.4%)	17 (17.2%)	27 (27.3%)	22 (24.2%)	14 (13.1%)	13 (12.1%)
*χ^2^p*-value			0.375	**0.307**	0.211	**0.0014**	**0.0121**
HWE *p-*value		0.907	1	0.325	0.663	0.978	0.452

*DRD2* rs1800497	CC	111 (65.3%)	64 (64.6%)	70 (70.7%)	57 (62.6%)	79 (73.8%)	67 (62.6%)
	CT	51 (30.0%)	30 (30.3%)	25 (25.3%)	30 (33.0%)	25 (23.4%)	33 (30.8%)
	TT	8 (4.7%)	5 (5.1%)	4 (4.0%)	4 (4.4%)	3 (2.8%)	7 (6.5%)
*χ^2^p*-value			0.972	0.300	0.712	**0.029**	0.574
HWE*p-*value		0.795	0.836	0.664	0.9998	0.841	0.584

*HTR2A* rs6313	CC	55 (39.0%)	27 (27.3%)	40 (40.4%)	40 (44.0%)	35 (32.7%)	24 (22.4%)
	CT	70 (49.6%)	54 (54.5%)	44 (44.4%)	36 (39.6%)	43 (40.2%)	58 (54.2%)
	TT	16 (11.3%)	18 (18.2%)	15 (15.2%)	15 (16.5%)	29 (27.1%)	25 (23.4%)
*χ^2^p*-value			**0.0307**	0.184	**0.0066**	**1.15 × 10** ^**−4**^	**2.97 × 10** ^**−5**^
HWE*p-*value		0.548	0.610	0.881	0.388	0.134	0.684

*COMT* rs4680	AA	34 (26.4%)	23 (23.2%)	36 (36.4%)	26 (28.6%)	27 (25.2%)	21 (19.6%)
	AG	62 (48.0%)	46 (46.5%)	45 (45.5%)	44 (48.4%)	47 (43.9%)	55 (51.4%)
	GG	33 (25.6%)	30 (30.3%)	18 (18.2%)	21 (23.1%)	33 (30.8%)	31 (29.0%)
*χ^2^p*-value			0.458	**0.022**	0.752	0.423	0.152
HWE*p-*value		0.908	0.806	0.838	0.960	0.470	0.929

*BDNF* rs6265	AA	2 (1.2%)	3 (3.0%)	1 (1.0%)	1 (1.1%)	7 (6.5%)	6 (5.6%)
	AG	39 (23.1%)	33 (33.3%)	31 (31.3%)	28 (30.8%)	31 (29.0%)	39 (36.4%)
	GG	128 (75.7%)	63 (63.6%)	67 (67.7%)	62 (68.1%)	69 (64.5%)	62 (57.9%)
*χ^2^p*-value			**0.0038**	0.0696	0.0953	**0.0017**	**8.17 × 10** ^**−6**^
HWE*p-*value		0.878	0.867	0.449	0.533	0.418	0.999

*GABRA2* rs279858	AA	52 (30.4%)	28 (28.3%)	32 (32.3%)	25 (27.5%)	32 (29.9%)	46 (43.0%)
	AG	78 (45.6%)	47 (47.5%)	53 (53.5%)	44 (48.4%)	59 (55.1%)	46 (43.0%)
	GG	41 (24.0%)	24 (24.2%)	14 (14.1%)	22 (24.2%)	16 (15.0%)	15 (14.0%)
*χ^2^p*-value			0.819	**9.63 × 10** ^**−4**^	0.670	**0.0023**	**8.83 × 10** ^**−5**^
HWE*p-*value		0.548	0.888	0.566	0.955	0.416	0.817

*CHRNA5* rs16969968	AA	20 (11.8%)	15 (15.2%)	8 (8.1%)	7 (7.7%)	22 (20.6%)	18 (16.8%)
	AG	74 (43.5%)	46 (46.5%)	44 (44.4%)	37 (40.7%)	57 (53.3%)	44 (41.1%)
	GG	76 (44.7%)	38 (38.4%)	47 (47.5%)	47 (51.6%)	28 (26.2%)	45 (42.1%)
*χ^2^p*-value			0.185	**1.25 × 10** ^**−30**^	**1.18 × 10** ^**−10**^	**1.52 × 10** ^**−27**^	**6.71 × 10** ^**−34**^
HWE*p-*value		0.954	0.985	0.566	0.955	0.955	0.817

Significant right-tail *p*-values (*p* < 0.05) for *χ^2^* statistics are marked in bold. *χ^2^p*-value, right-tail *p*-values for *χ^2^* statistics. HWE *p*-value, *p*-values for Hardy–Weinberg equation.

## Data Availability

The data used to support the findings of this study are available from the corresponding author upon request.
